# The Hospital World

**Published:** 1911-04-29

**Authors:** 


### The Hospital World.

### Eighty Years' Service at the Salford Royal Hospital.

The resignation of Mr. Andrew Boutflower, the senior Honorary Surgeon of Salford Royal Hospital, was accepted by the Board last week. Mr. Boutflower was appointed to the office of Honorary Surgeon in 1870, in succession to his father, who then resigned, after upwards of forty-three years of service, which began with the foundation of the hospital in 1827. The following is his letter to the Chairman of the Board :—

"Dear Mr. Pilkington,—You are probably aware that I have for some time contemplated resigning my office of Surgeon to the Salford Royal Hospital; I have hitherto delayed taking this step for reasons which I need not recount. The opening of the new wards suggests to me that my retirement should take place at that date. I therefore ask you to convey my resignation to the Board of Management, to take effect when the new wards come into use. I should like to record that I was appointed to this office at the end of the year 1870, after serving for two years the office of House Surgeon to the Manchester Royal Infirmary.

"My father resigned after nearly forty-four years' service, and I was at once appointed in his stead, so that we have been permitted between us to serve the poor and needy at the hospital for more than eighty-four years. I am happy also to record the complete harmony which has always existed between myself and the Board of Management, and between myself and my hospital colleagues. I need hardly add that I shall always have the interests of the hospital in my heart, and shall at all times be delighted to render it any service in my power. Yours most faithfully, (signed)

Andrew Boutflower."

### An Example of Personal Service.

By the death of Mr. J. T. Belfrage, of Putney, the Royal Hospital for Incurables on West Hill, has lost a devoted friend. Mr. Belfrage served for some years on the Board of Management of the Hospital. He was also a member of the House Committee and of the Finance Committee. His attendances were many at these meetings. Mr. Belfrage was also a quiet and unostentatious visitor. Though sometimes racked with sciatica, he would walk up the Hill to the hospital with presents for the invalids and he would sit and talk with them and hear all about their ailments and their troubles, making friends of all with whom he came in contact. He had an interest in the Hospital Farm, and many were the hours he gave to the improvement of the hospital poultry stock. He interested himself in securing new friends and new subscribers for the hospital and he had a personal regard for the officers of the institution; he was good enough to consider himself a friend and a fellow-worker. On occasions he could be critical when circumstances warranted it, but he was always fair. He thought nothing of writing many letters to friends and acquaintances, whose names he considered ought to appear in the list of subscribers to the institution in which he took so keen an interest himself.

### A Former Messenger Boy's Bequests to Hospitals

The Hull Royal Infirmary and the Victoria Hospital for Poor Sick Children, Hull, will count among their most interesting bequests of this year the £200 and £100 which respectively they have received by the will of the late Mr. George Thomas Appleyard. Mr. Appleyard, who left over £50,000, was at his death managing director of a large firm of hardware merchants, into the service of whom he had entered some sixty-five years ago as messenger boy. Besides the two hospitals mentioned, other Hull charities have benefited by his will.

### The late Lord Airedale.

Speaking the other day at the annual meeting of the Leeds General Infirmary, Mr. Charles Lupton, the hospital's energetic treasurer and chairman of the weekly board, made a very interesting statement on Lord Airedale's work, which we quote : He was never a member of the board, I believe—at any rate, he has never been on the board during the time I have been here. Pressure of business and other matters kept him from it, but he has always been one of our best friends. He inherited a great interest in the Infirmary from his father, and took quite a pride in the fact that his father was the chairman of the Building Committee which built this hospital, and that he was largely responsible for the ambitious lines upon which it was planned. I have been told that there were occasions when several members of the committee were doubtful about planking down all the money they could get to build the hospital we see, but Mr. Kitson was always ready to go on, and he said he felt quite sure that if they built the best thing it would be the best for the city. That was the father of Lord Airedale, and Lord Airedale inherited the same interests and the same pluck, and he was always ready to come here and help in any way he could. It was only two months ago that he took me to see what he called the greatest treasure which he possessed, and that was the mallet and trowel given to his father when the foundation stone of this building was laid. During the last year we have had some notable marks of his personal interest; first of all in the very difficult question we had to solve with regard to our own working so far as it was connected with the working of other medical institutions. He took part in our conferences, and was most keen that we should come to a satisfactory result, and I think he helped very materially in attaining that result. Whilst he was abroad I had several letters from him on the subject, and it is a very great loss to us that he has died at this time. The Infirmary has had many good friends, but I do not think it has ever had a better one than Lord Airedale.

### The late Librarian of Guy's.

Old Guy's men will hear with regret the news that Mr. Mills, the Librarian of the Wills Library, died suddenly recently. Mr. Mills was originally an Army schoolmaster, and was chosen out of a large number of applicants to fill the post which for many years he occupied so ably that he was looked upon as being as much an institution as the Library itself. Always courteous and willing, and with that stock of unlimited patience which a librarian in a reference library needs to be provided with, he possessed also a rare and scholarly knowledge of medical bibliography, which rendered his advice and assistance of special value to research workers. The writer well remembers the intimate acquaintance which Mr. Mills showed on more than one occasion with the somewhat peculiar catalogue of old English editions of Bullein, Halle, and Lister, the nucleus of the old Physical Society's original collection. Mr. Mills was perhaps the most popular and best-known figure at Guy's. Everyone knew him, everyone liked him, and those who came to know him more intimately and who gained his friendship, learned to know that his popularity was gained through solid worth and not mere trimming. During the last few years his health was very bad and he was plagued with chronic bronchitis, which did not however prevent him from following his duties in the library. The end came suddenly and unexpectedly one afternoon when he had sat down to read after luncheon. His loss will be felt by many "years" of Guy's men.

